# Silica induces NLRP3 inflammasome activation in human lung epithelial cells

**DOI:** 10.1186/1743-8977-10-3

**Published:** 2013-02-12

**Authors:** Paul M Peeters, Timothy N Perkins, Emiel F M Wouters, Brooke T Mossman, Niki L Reynaert

**Affiliations:** 1 Department of Respiratory Medicine, Maastricht University Medical Centre+ (MUMC+), Maastricht University, Maastricht, The Netherlands; 2 Department of Pathology, University of Vermont College of Medicine, 89 Beaumont Avenue, Burlington, VT, 05405, USA

**Keywords:** Silica, NLRP3 inflammasome, Caspase-1, IL-1β, HMGB1, bFGF

## Abstract

**Background:**

In myeloid cells the inflammasome plays a crucial role in innate immune defenses against pathogen- and danger-associated patterns such as crystalline silica. Respirable mineral particles impinge upon the lung epithelium causing irreversible damage, sustained inflammation and silicosis. In this study we investigated lung epithelial cells as a target for silica-induced inflammasome activation.

**Methods:**

A human bronchial epithelial cell line (BEAS-2B) and primary normal human bronchial epithelial cells (NHBE) were exposed to toxic but nonlethal doses of crystalline silica over time to perform functional characterization of NLRP3, caspase-1, IL-1β, bFGF and HMGB1. Quantitative RT-PCR, caspase-1 enzyme activity assay, Western blot techniques, cytokine-specific ELISA and fibroblast (MRC-5 cells) proliferation assays were performed.

**Results:**

We were able to show transcriptional and translational upregulation of the components of the NLRP3 intracellular platform, as well as activation of caspase-1. NLRP3 activation led to maturation of pro-IL-1β to secreted IL-1β, and a significant increase in the unconventional release of the alarmins bFGF and HMGB1. Moreover, release of bFGF and HMGB1 was shown to be dependent on particle uptake. Small interfering RNA experiments using siNLRP3 revealed the pivotal role of the inflammasome in diminished release of pro-inflammatory cytokines, danger molecules and growth factors, and fibroblast proliferation.

**Conclusion:**

Our novel data indicate the presence and functional activation of the NLRP3 inflammasome by crystalline silica in human lung epithelial cells, which prolongs an inflammatory signal and affects fibroblast proliferation, mediating a cadre of lung diseases.

## Background

Crystalline silica (SiO_2_) is the second most common mineral in the earth’s crust and is the major component of sand, rock and mineral ores. In occupational and environmental settings, microscopic mineral particles in airborne dust, generated by wind, manufacturing or demolition are inhaled and can get taken up by epithelial cells lining the respiratory tract, initiating and sustaining inflammatory responses at high concentrations. Prolonged exposure in the workplace may lead to the development of silicosis, which can be irreversible and is characterized by the development of progressive pulmonary fibrosis [1]. Crystalline silica exists in many different polymorphs, but those of particular concern are the naturally occurring polymorphs quartz, cristobalite and tridymite.

The Nacht Domain- Leucine-Rich Repeat-, and PYD-containing Protein 3 (NLRP3) inflammasome has recently been recognized as an innate immune signaling receptor important in mediating cell responses to various endo- and exogenous signals [2-4]. This multi- protein platform interacts with the apoptosis-associated speck-like protein PYCARD/ASC, which contains a caspase-1 recruitment domain. Detection of a danger signal such as crystalline silica particles in macrophages, activates the inflammasome complex leading to binding and activation of pro-caspase-1, which in turn causes the cleavage and secretion the of pro-inflammatory cytokines interleukin – 1 beta (IL – 1β) and interleukin – 18 (IL – 18) to their active forms [5-7]. IL-1, a very potent and pivotal mediator of inflammatory responses induced by silica exposure [8,9] affects virtually all tissue types and has been implicated in the pathophysiology of human and experimental silicosis [10,11]. Additionally, caspase-1 expression and activation is required for unconventional secretion of IL-33, IL-1α, basic fibroblast growth factor (bFGF) and high mobility group box 1 (HMGB1) although these proteins are not substrates of caspase-1 [12-15].

Until recently, it was assumed that the initial responses of lung tissue to silica were orchestrated mainly by cells of the innate immune system, such as monocytes, macrophages, neutrophils, and dendritic cells. Purified primary human monocyte-derived macrophages, peripheral blood mononuclear cells, and mouse bone marrow-derived macrophages release IL-1β after inflammasome activation by silica [6,7]. NLRP3 knockout mouse models develop reduced pulmonary inflammation and less abundant collagen deposition compared to wild-type animals following silica administration [5]. Importantly, in these studies the localization of the active inflammasome in lung tissue was not shown.

Non-myeloid cells can also be important guardians for the detection of danger signals, fulfilling tasks usually performed by resident macrophages. Many groups have demonstrated NLRP3 presence and functional activity in non-myeloid cells by various agonists. For example, nanoparticles activate the NLRP3 inflammasome, leading to IL-1β secretion in primary human keratinocytes [16]. Recently, expression of the NLRP3 inflammasome in human airway epithelium following *in vivo* particulate matter exposure has been shown although its functional significance in lung disease was unknown [17]. Because the lung epithelial surface is one of the largest primary barriers to environmental exposures and the initial site of impingement of respirable silica, we hypothesized that bronchial epithelial cells are an important target of inflammasome activation. This activation may fuel cross-talk between neighboring fibroblasts, endothelial cells, as well as cells of the immune system which in turn release secondary mediators and initiate or mediate fibrogenesis.

## Materials and methods

### BEAS-2B cell culture

Non-tumorigenic human bronchial epithelial cells (Ad12-SV40 immortalized) BEAS 2B (ATCC, Manassas, VA) were grown and maintained in Dulbecco's Minimal Essential Medium (DMEM)/F12 containing 10% Fetal Bovine Serum (FBS) (CellGro^®^ Mediatech inc, Manassas, VA), with penicillin (50 U/ml), streptomycin (100 μg/ml) (Invitrogen, Carlsbad, CA), hydrocortisone (100 μg/ml), insulin (2.5 μg/ml), transferrin (2.5 μg/ml) and selenium (2.5 μg/ml) (Sigma, St. Louis, MO). Culture flasks and plates (BD, Franklin Lakes, NJ) were pre-coated with a mixture of fibronectin (Sigma, St. Louis, MO) (0.01 mg/ml), bovine collagen type I (0.03 mg/ml) (Invitrogen, Carlsbad, CA) and bovine serum albumin (0.01 mg/ml) (Sigma, St. Louis, MO), in DMEM/F12 media for 24 h at 37°C . Prior to exposures, medium was aspirated and replaced with reduction medium containing 0.5% FBS. In selected experiments BEAS-2B cells were primed with 5 μg/mL LPS for 4 h prior to silica exposure. Particle uptake was blocked by administration of 0.5 μg/mL cytochalasin D for 1h prior to silica exposure.

### NHBE cell culture

Primary normal human bronchial epithelial cells (NHBE-17917, Lonza, Clonetics^®^) were cultured and maintained in BEGM^®^ (Lonza, Clonetics^®^. (Switzerland)) according to the manufacturer’s protocol.

### MRC-5 cell culture

The MRC-5 (CCL-171) cell line, a human fetal lung fibroblast cell line, was obtained from the ATCC and maintained in Eagle's Minimum Essential Medium (Gibco) supplemented with L- Glutamine (200 mM, Invitrogen), 100 U/ml penicillin, 100 μg/ml streptomycin, and 0.5% heat-inactivated fetal calf serum (Gibco) and non-essential amino acids (MP Biomedicals). For addition of conditioned media, MRC-5 cells were serum starved for 24 h in Eagle's Minimum Essential Medium (Gibco) supplemented with L- Glutamine (200 mM, Invitrogen), 100 U/ml penicillin, 100 μg/ml streptomycin, and 0.5% heat-inactivated fetal calf serum (Gibco) and non-essential amino acids (MP Biomedicals).

### THP-1 cell culture

THP-1 cells obtained from ATCC were grown in RPMI 1640 medium containing 10% fetal bovine serum with penicillin (50 U/ml), streptomycin (100 μg/ml) and 2 mM L-glutamine at 37°C. Ten ng/mL PMA was used to differentiate THP-1 cells for 24-36 h prior to experiments.

### Particle exposures

Cristobalite silica particles (C & E Mineral Corp., King of Prussia, PA) were UV-irradiated over night to inactivate possible contaminating endotoxin. Silica particle suspensions (1mg/mL) were sonicated for 15 min, aspirated 5 times through a 23 gauge needle and added to cell cultures. Throughout the studies presented in this paper, we utilized several particle doses based on their surface area characteristics and toxicity [18]. Glass beads (1–4 μm diameter), obtained from Particle Information Services, Inc. (Kingston,WA) were incorporated as a negative control based on particle surface area metrics.

### siRNA mediated knock down in BEAS-2B and THP-1 cells

siRNA against NLRP3 (ON-TARGET plus SMARTpool L-017367–00-0005: GGAUCAAACUACUCUGUGA, UGCAAGAUCUCUCAGCAAA, GAAGUGGGGUUCA GAUAAU, and GCAAGACCAAGACGUGUGA) and the ON-TARGET plus GAPDH Control Pool (human); were purchased from Dharmacon, (Thermo Scientific, USA). In BEAS-2B cells siRNA transfections were performed with Lipofectamine 2000 and 100nM siRNA 24 h prior to stimulations. THP-1 cells were differentiated with 10 ng/mL PMA over night after which 100nM siRNA was transfected, using Lipofectamine 2000.

### RNA isolation

Total RNA was prepared using an RNeasy^®^ Plus Mini Kit according to the manufacturer’s protocol (Qiagen, Valencia, CA) and 1 μg was reverse-transcribed with random primers using the AMV Reverse Transcriptase kit (Pro-mega, Madison, WI). Primers for human NLRP3 were fw:GAT CTTCGCTGCGATCAACAG and rev:CGT GCATTATCTGAACCCCAC. Primers used to determine caspase-1 mRNA levels were fw:TTTCCGCAAGGTTC GATTTTCA and rev:GGCATCTGCGCTCTACCATC*.* HPRT was used as the housekeeper gene (fw:GACCGGT TCTGTCATGTCG, rev:ACCTGGTTCATCATCACTAA TCAC). Primer sequences were taken from Primerbank. Fold changes in the expression of genes of interest were calculated using the ΔΔCt method. Duplicate assays were performed with all samples.

### Western blots

Cells were lysed in a buffer containing 20 mM Tris, 150 mM NaCl, 1% Nonidet P-40, 1 mM DTT, 1% Protease Inhibitor Cocktail and 1% Phosphatase Inhibitor Cocktail. Total protein content was determined by the Bio-Rad DC Protein Assay kit (Bio-Rad, Hercules, CA), and 20-40 μg of protein was loaded onto polyacrylamide gels. After transfer of proteins to a nitrocellulose membrane, primary antibodies against caspase-1 (Santa Cruz Biotechnology, Santa Cruz, CA), β-actin (Cell Signaling Technologies), IL-1β (Cell Signaling Technologies), NLRP3 (Santa Cruz Biotechnology) or HMGB1 (Cell Signaling Technologies) were applied. After three washes with TBS-Tween, HRP conjugated secondary antibodies were detected by chemiluminescence according to the manufacturer’s instructions. Densitometry was performed on scanned immunoblot images using the QuantityOne software (Bio-Rad Laboratories). Supernatants were concentrated using Amicon Ultra centrifugal filters (Millipore) and analyzed as described above.

### Caspase-1 activity assay

Caspase-1 activity was measured using a caspase-1 activity kit (R & D) following the manufacturer’s protocol.

### ELISA

The levels of IL-1β (Biolegend) and bFGF (Biolegend) in cell culture media were measured using human ELISA kits. HMGB1 was determined using a direct ELISA protocol. The primary antibody (ab18256 Abcam) was used at a 1/1000 dilution for 16 h at 4°C. The secondary antibody was a biotin-conjugated swine anti-rabbit used at a 1/1000 dilution. A blocking step was performed using 5% BSA for 1 h. Concentrations of IL-1β, bFGF and HMGB1 were established via extrapolation from a standard curve of the appropriate recombinant protein.

### Proliferation assay

BEAS-2B cells were treated with silica suspensions for 24 h. Supernatants were collected and centrifuged to remove suspended particulates after which conditioned media was added to MRC-5 cells at a 1 to 4 ratio. At indicated time points, MRC-5 cells were fixed with ice cold 70% ethanol for 5 min. After aspiration, 5N NaOH was added followed by DNA content measurement at 260 nm.

### Statistical analyses

All experiments were performed 3 times. Data were analyzed by one-way analysis of variance (ANOVA) using the Student Neuman-Keul’s test to adjust for multiple pair-wise comparisons between treatment groups, or the Student's *t* test where appropriate. Data from multiple experiments were averaged and expressed as mean values ± SEM. Differences with p-values < 0.05 were considered statistically significant.

## Results

### Cristobalite silica particles induce increased mRNA levels and enzymatic activity of caspase-1 in primary human bronchial epithelial cells

**Figure 1:**
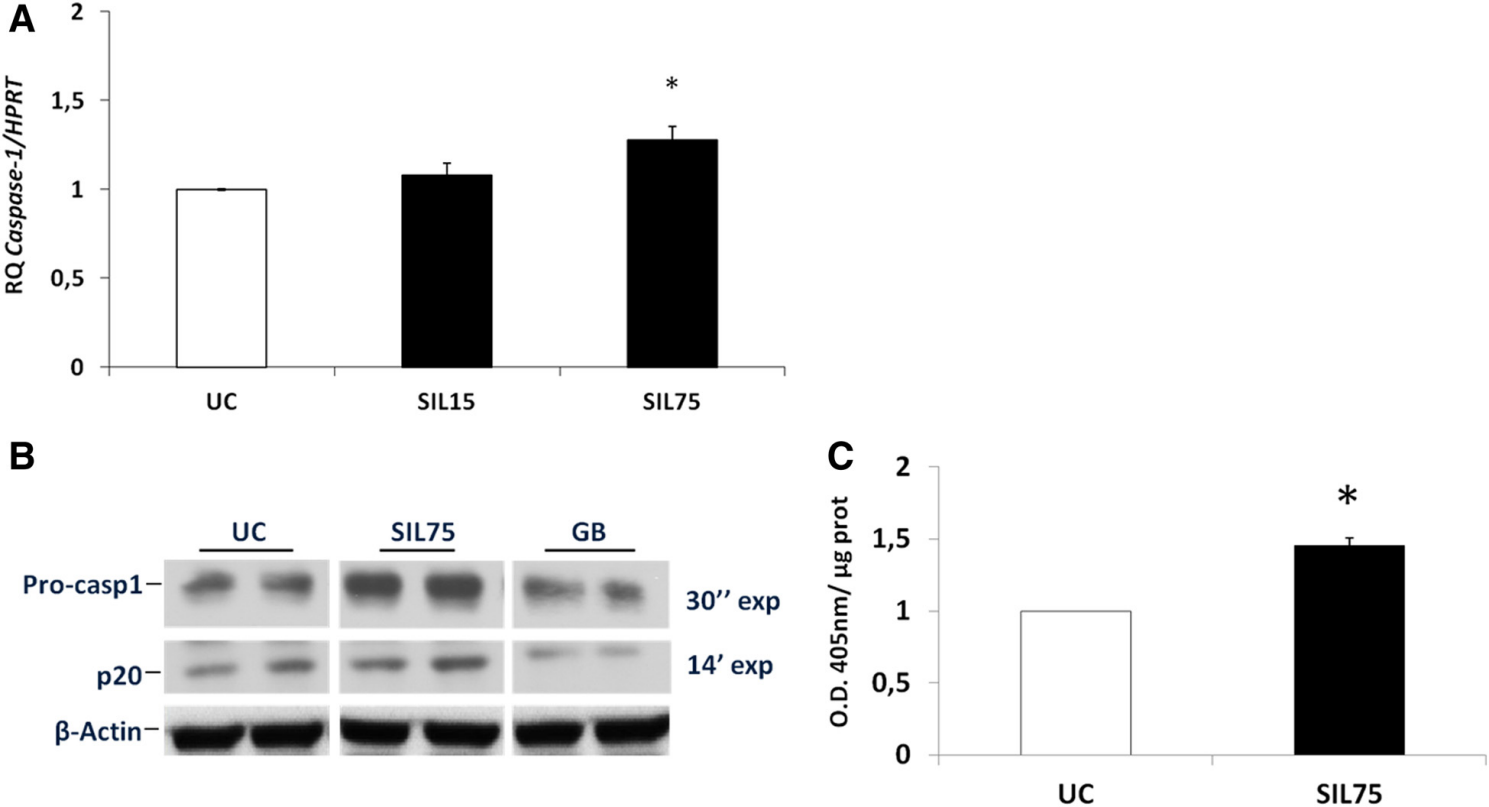
**Cristobalite silica increases caspase-1 transcript levels and caspase -1 cleavage products in primary human bronchial epithelial cells. A. **Caspase-1 mRNA levels in primary normal human bronchial epithelial (NHBE) cells after 24 h exposure to 15 (SIL15) and 75 × 10^6^μm^2^/cm^2^ (SIL75) cristobalite silica, expressed as mean fold change ± SEM (indicated by error bars) with *p-value <0.05 compared to unexposed control cells (UC) and normalized to the housekeeping gene *HPRT *(N= 3). **B. **Western blot analysis of caspase-1 cleaved products was conducted on whole cell lysates of NHBE cells after 24 h of exposure. p20 subunits were detected after a longer exposure compared to pro-caspase-1 (14 min versus 30 s). Glass beads (GB) and unexposed cells (UC) were included as controls, and β-actin was used as a loading control. **C**, Caspase-1 enzymatic activity was assayed in cell lysates of NHBE cells exposed to the indicated silica concentrations for 24 h with. Caspase-1 activities are expressed as arbitrary units (O.D. 405 nm/μg protein) with *p-value <0.05 compared to UC.

Based on an available microarray dataset in the BEAS-2B cell line and primary human bronchial epithelial (NHBE) cells [18] we identified a group of genes related to the inflammasome as well as to the pro-inflammatory cytokine, IL-1β with significantly altered expression levels following 24 h exposure to cristobalite silica (Table 1). As described before, we worked with silica at 75 × 10^6^μm^2^/cm^2^ on NHBE cells, a concentration with an approximate 25% cytotoxicity that allowed us to investigate the maximum inflammatory and possible fibrogenic effects of these mineral particles [18]. We next performed RT-PCR to investigate the quantitative mRNA expression levels of caspase-1 in NHBE cells. Figure 1A shows a significant increase of caspase-1 mRNA levels in primary human bronchial epithelial cells when exposed to 75 × 10^6^μm^2^/cm^2^ (SIL75). NRLP3 mRNA levels were not enhanced in NHBE cells exposed to silica (data not shown). To investigate whether silica exposure in addition to increasing transcript levels of inflammasome-related genes would lead to the activation of pro-caspase-1, caspase-1 cleavage was determined by Western blotting. Figure 1B shows increases in protein levels of the functional p20 subunit of caspase-1 in NHBE whole cell lysates after treatment with SIL75 for 24 h. Importantly, glass beads (GB, negative control), which are dense amorphous microspheres with decreased surface reactivity, did not induce increased levels of caspase-1 cleaved subunits. A caspase-1 activity assay confirmed its increased activation in NHBE cells exposed to SIL75 (Figure 1C).

**Table 1:** Selection of NLRP3 inflammasome and IL-1 family member related genes

**BEAS-2B**		**mRNA**
**Gene symbol**	**Gene name**	**Fold Change ↑**
*IL1A*	Interleukin 1, alpha	6,2*
*IL1RL1*	Interleukin 1 receptor-like 1	5,4
*IL1B*	Interleukin 1, beta	3,6*
*NLRP1*	NLR family, pyrin domain containing 1	3,4
*NLRP3*	NLR family, pyrin domain containing 3	2,5*
*IL1RL2*	Interleukin 1 receptor-like 2	2,3
*IL18R1*	Interleukin 18 receptor 1	2,2
*IL1RAP*	Interleukin 1 receptor accessory protein	2,2
NHBE		
	Gene name	Fold Change ↑
*IL1F9*	Interleukin 1 family, member 9	14,0
*TXNIP*	Thioredoxin interacting protein	9,7
*IL1F5*	Interleukin 1 family, member 5 (delta)	6,0
*IL32*	Interleukin 32	5,5
*IL1RL1*	Interleukin 1 receptor-like 1	3,5
*CASP1*	Caspase 1 (interleukin-1 beta convertase)	2,1*
*bFGF*	Fibroblast growth factor 2 (basic)	2,1
*IL1A*	Interleukin 1, alpha	2,1

For each cell type a selection of genes was collected from previously described microarray data set [18]. All transcripts are involved in inflammasome and IL-1 immune- cytokine responses.

* = confirmed by qRT-PCR.

### Cristobalite silica particles induce increased NLRP3 mRNA levels as well as elevated levels of cleaved caspase-1 subunits

**Figure 2:**
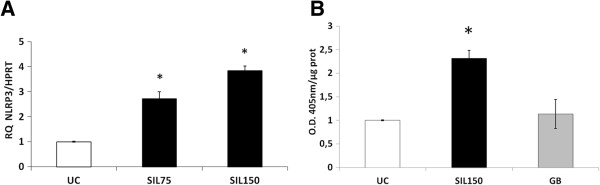
**Crystalline silica increases NLRP3 mRNA levels and caspase-1 cleavage products in BEAS-2B cells. A. **Bar chart represents relative quantity of *NLRP3* mRNA levels after 24 h exposure to 75 × 10^6^μm^2^/cm^2 ^(SIL75) and 150 × 10^6^μm^2^/cm^2 ^(SIL150) of cristobalite silica in BEAS-2B cells. *p-value <0.05 compared to UC. **B. **Caspase-1 enzymatic activity was assayed in cell lysates of BEAS-2B cells exposed to the indicated silica concentrations for 24 h. Glass beads (GB) and unexposed cells (UC) were included as controls. Caspase-1 activities are expressed as arbitrary units (O.D. 405 nm/μg total protein) with *p-value <0.05 compared to UC.

In BEAS-2B cells we first performed additional assays over a range of concentrations of cristobalite silica to determine toxicity (Additional file 1: Figure S1). For further experiments, silica concentrations were used that were toxic, causing an acceptable maximum of 25% cell death. In contrast to NHBE cells, cristobalite silica was found to increase levels of NLRP3 mRNA dose dependently in BEAS-2B cells, after 24 h exposure (Figure 2A). In agreement with data in NHBE cells, we confirmed silica-induced caspase-1 activity in BEAS-2B cells following SIL150 exposure. Glass beads also did not affect caspase-1 activity significantly in these cells (Figure 2B).

### Cytokine maturation and alarmin secretion in BEAS-2B cells in response to cristobalite silica

**Figure 3:**
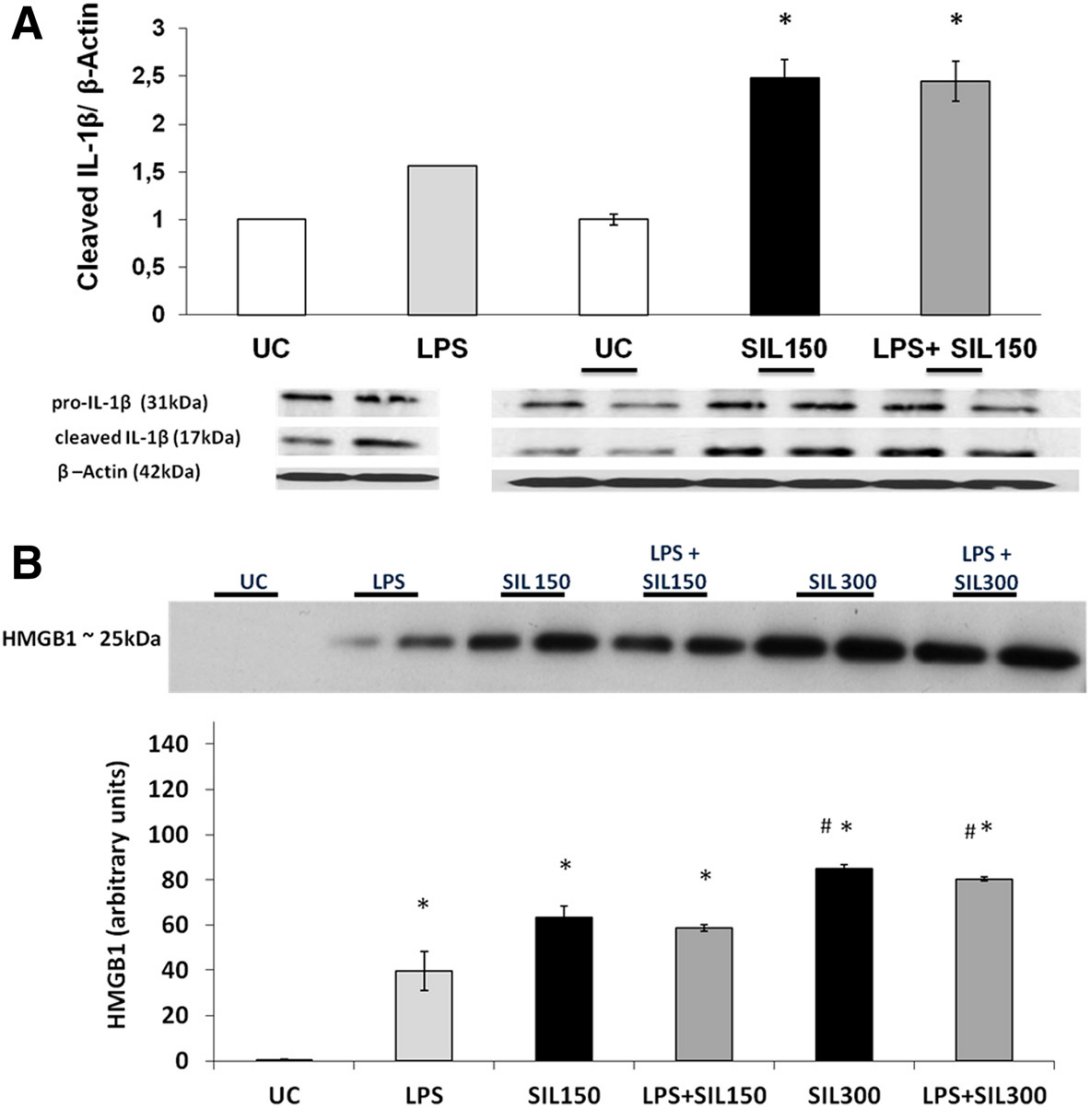
**Silica augments processing and secretion of inflammatory mediators and DAMPs in bronchial epithelial cells. A. **Western blot analysis was conducted on whole cell lysates of BEAS-2B cells to detect cleaved IL-1β products, after 24 h of silica exposure with or without priming with 5μg/mL LPS for 4h. Histogram represents results of densitometric analysis of cleaved IL-1β normalized to β-actin. **B. **Western blot analysis of HMGB1 release in the concentrated SN from silica exposed BEAS-2B cells. Histogram represents results of semi-quantitative densitometric analysis of the HMGB1 signal expressed as arbitrary units. Data are presented as mean fold change ± SEM with * p-value <0.05 compared to UC and # p-value <0.05 compared to SIL150 or LPS+SIL150.

Classically, inflammasome complex assembly, accompanied by caspase-1 activation leads to cleavage of pro-inflammatory IL-1β into its active form that is secreted. Interleukin (IL)- 1β is produced by a variety of cell types in response to inflammatory stimuli such as lipopolysaccharide (LPS) [19]. In some studies it was shown that pro-IL1β mRNA levels first need to be elevated by a TLR-dependent priming stimulus, such as LPS before enhanced release of mature IL1β can be detected by an inflammasome stimulus. We therefore investigated whether silica-induced release of IL-1β requires LPS-priming in human bronchial epithelial cells, and whether LPS alone could trigger IL-1β maturation and release in BEAS-2B cells. LPS administered to BEAS-2B cells showed only a moderate increase in detectable levels of the cleaved IL1β subunits. Cells exposed to SIL150 demonstrated significantly increased intracellular maturation of pro-IL1β. LPS priming, followed by silica exposure, did not further enhance silica-induced maturation of IL-1β (Figure 3A). We also investigated whether silica affected the release of alarmins linked to caspase-1 activation. A dose-dependent increase in HMGB1 levels in culture medium supernatants (SN) was detected following cristobalite exposure. Although LPS on its own induced a substantial increase of HMGB1 in media, LPS priming followed by silica exposure did not further augment HMGB1 levels in the medium compared to silica exposure alone (Figure 3B). We furthermore previously reported a significant dose-dependent release of bFGF from BEAS-2B cells following exposure to SIL150 for 24 h [18].

### The NLRP3 inflammasome mediates cristobalite silica-induced inflammatory danger signaling

**Figure 4:**
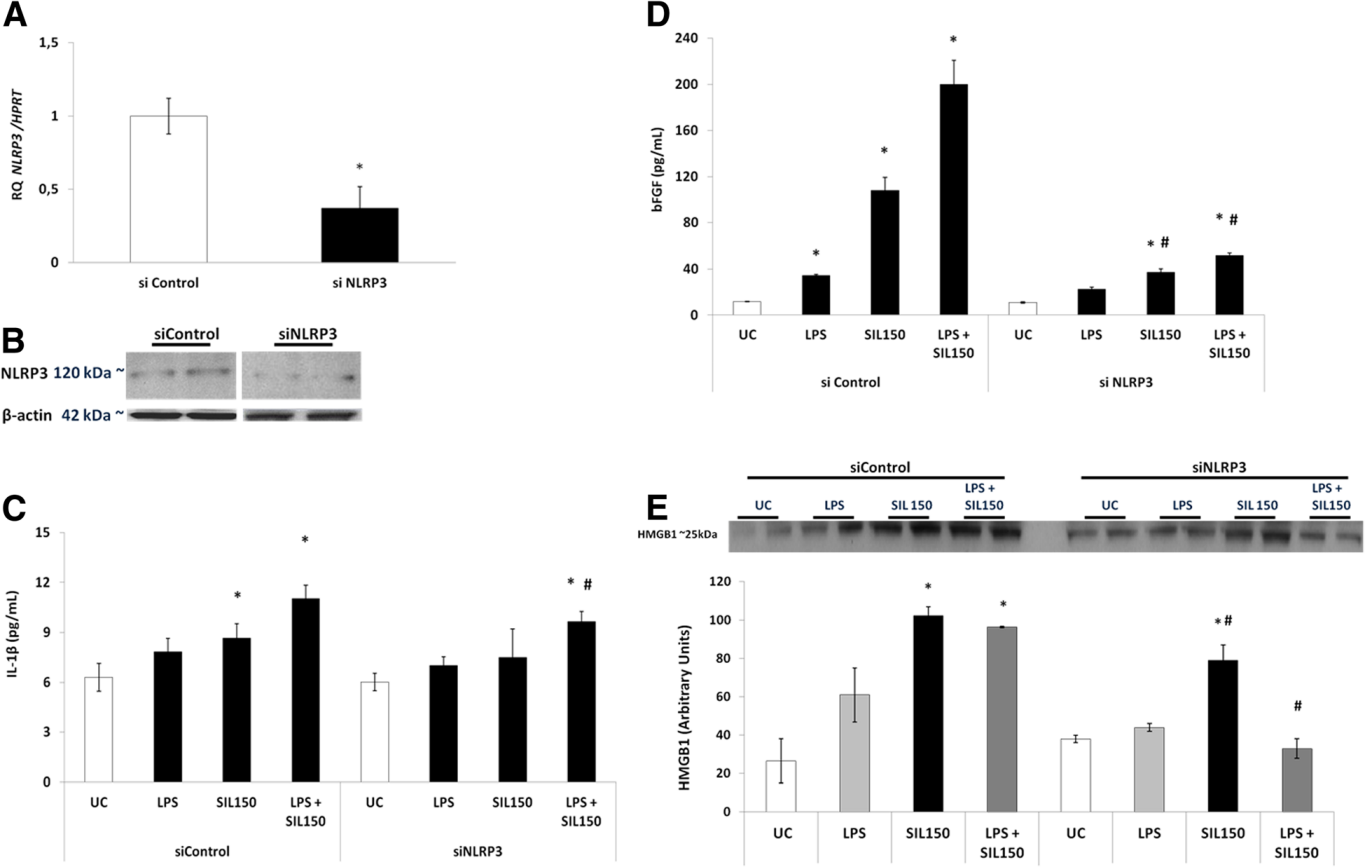
**Canonical and non-canonical release of inflammatory mediators and DAMPs is regulated by NLRP3. **BEAS-2B cells were transfected with control (siControl) or NLRP3 siRNA (siNLRP3). Experiments were performed 24 h after siRNA transfection. **A. **Knockdown of NLRP3 mRNA levels by siRNA. **B. **Western blot analysis of whole cell lysates for the level of NLRP3 expression. Actin was used as a loading control. ELISA was performed on concentrated medium SN for detection of secreted IL-1β (**C**) and bFGF (**D**) 24 h post-transfection. The effect of LPS priming (5 μg/mL) was also investigated. **E. **Western blot analysis, to detect influence of NLRP3 knockdown on HMGB1 levels was conducted on concentrated medium supernatants. Histogram represents semi-quantitative densitometric analysis of HMGB1 expressed as arbitrary units. Data are presented as mean fold change ± SEM with *p-value <0.05 compared to UC, and # p-value <0.05 compared to the same treatment in the siControl setting.

In order to investigate whether cristobalite silica-induced release of IL1β, HMGB1 and bFGF factors is mediated by the inflammasome, we performed RNAi-mediated knockdown of the NLRP3 gene. Transfection of NLRP3 siRNA in BEAS-2B cells was highly efficient in reducing NLRP3 mRNA levels >50% as shown in Figure 4A. In parallel, the protein abundance of NLRP3 was reduced by transfection of NLRP3 siRNA (Figure 4B). As previously shown in Figure 3A, increased levels of IL-1β at SIL150 and an absence of a synergistic effect of LPS priming in the siRNA control group were found. Also, LPS priming alone did not induce significantly higher levels of IL-1β in the medium. The absence of effect of NLRP3 knock-down on basal IL-1β levels could be due to the activity of other enzymes including lysosomal enzymes and MMPs that can cleave IL-1β or the residual level of NLRP3. Although priming with LPS still significantly enhanced IL-1β levels in response to silica in the NLRP3 siRNA transfected cells, these levels were significantly attenuated compared to the control siRNA transfected cells (Figure 4C). As alternative IL1β processing enzymes mentioned above are known to be activated by silica and LPS, these alternative routes of processing could explain this attenuated, but not abrogated response in NRLP3 siRNA conditions. Increased levels of secreted bFGF by SIL150 could be further augmented by priming bronchial epithelial cells with LPS under control siRNA conditions, although not statistically significant. In all these conditions, bFGF levels were drastically attenuated after NLRP3 siRNA transfection, indicating a pivotal role for the inflammasome in bFGF release as seen in Figure 4D. The increased HMGB1 levels in medium caused by SIL150 alone, as well as after pre-treatment with LPS were also significantly reduced in the NLRP3 siRNA group (Figure 4E).

### Comparison of inflammasome-dependent mediator release from bronchial epithelial cells versus macrophages in response to silica

We conducted parallel experiments in BEAS-2B and THP-1 macrophages using the same dose of silica to investigate IL-1β levels and the effect of LPS priming on these cell types comparatively. Basal levels of secreted IL-1β are higher in THP-1 cells compared to BEAS-2B (28.3 ± 2.7 vs 6.29 ± 0.5 pg/ml respectively). Silica induced IL-1β levels in BEAS-2B cells to 8.67 ± 0.5 pg/ml. In THP-1 cells silica alone also induced IL-1β release, to a level of 191.0 ± 6.7 pg/ml, about 22-fold higher compared to in BEAS-2B. LPS had no significant effect by itself on IL-1β levels in either cell type. In BEAS-2B there was no synergistic effect of priming with LPS prior to silica treatment, whereas this was the case in THP-1 cells. HMGB1 levels in culture medium were also approximately double in THP-1 cells compared to BEAS-2B cells, under baseline conditions as well as after silica stimulation. bFGF levels on the other hand were comparable in unstimulated cells, but induction was greater in bronchial epithelial cells (Additional file 2: Table S1). The release of IL1β, HMGB1 and bFGF induced by silica was also NLRP3-dependent in THP-1 cells as indicated in Additional file 3: Figure S2.

### Uptake dependence of inflammasome signaling

**Figure 5:**
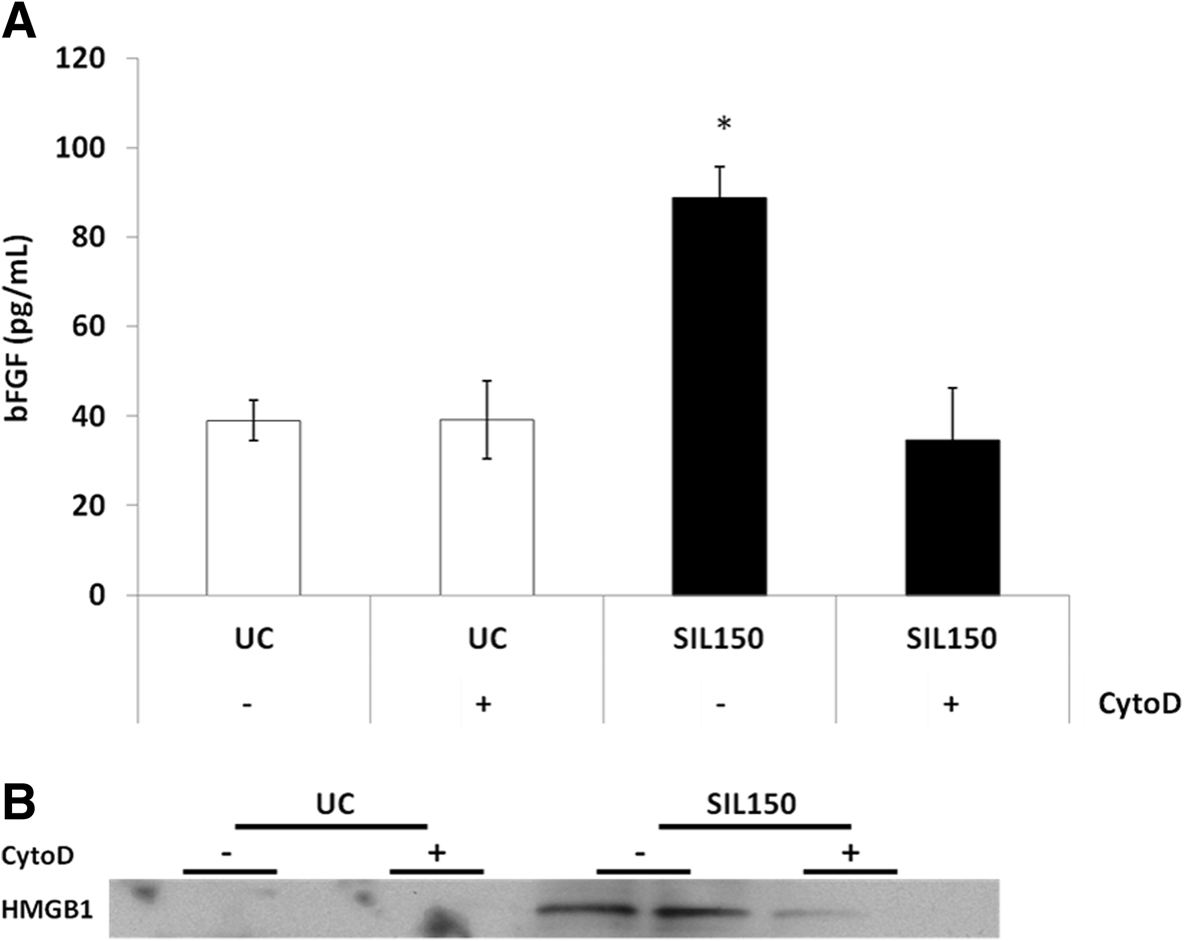
**bFGF and HMGB1 release are particle uptake dependent. **ELISA was performed on medium of BEAS-2B either or not pretreated with 0.5 μg/mL cytochalasin D for 1h measuring levels of bFGF release (**A**). **B. **Western blot analysis showing the effect of cytochalasin D on the release of HMGB1 in the medium following cristobalite silica exposure.

To investigate if particle uptake was pivotal to silica-induced inflammasome activation and downstream readouts, actin polymerization was blocked using 0.5 μg/mL cytochalasin D for 1 h. Cytochalasin D administration for 1 h prior to silica exposure markedly attenuated increased bFGF and HMGB1 secretion (Figure 5A and 5B, respectively). Importantly, a loss of cell viability in the presence of cytochalasin D was not detected (data not shown).

### Inflammasome activity in bronchial cells is linked to proliferative effects on MRC-5 fibroblasts and is dependent on particle uptake

**Figure 6:**
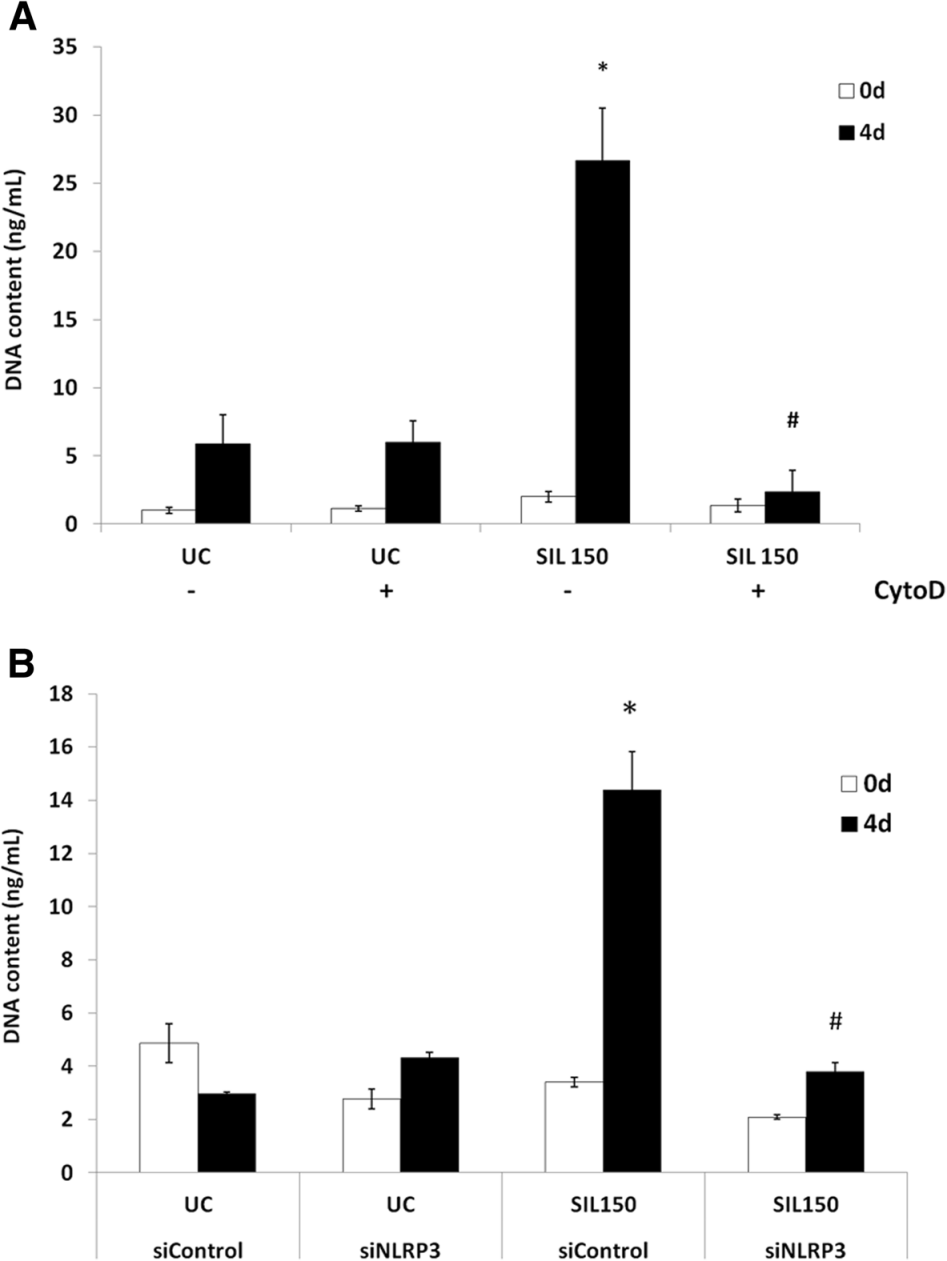
**Fibroblast proliferation is affected by exposure to bronchial epithelial cell conditioned media. **Conditioned BEAS-2B media was added to a monolayer of MRC-5 fibroblast cells and DNA content was measured up to 4 days to give an indication of proliferation. **A. **MRC-5 proliferation is impacted by inhibition of particulate uptake in BEAS-2B cells.** B. **Fibroblast proliferation after exposure to conditioned media of siRNA transfected BEAS-2B cells. *indicates a statistically significant difference in DNA content compared to UC at each time point, whereas # p<0.05 indicates significant difference compared to the positive control. N= 3 for each time point in each media condition.

To elucidate whether the inflammasome mediated paracrine microenvironment signaling in our cell culture model, conditioned medium of BEAS-2B cells after exposure to silica was tested for fibroblast proliferative capacity. Indeed, the conditioned medium of silica-exposed epithelial cells drastically increased the DNA content of MRC-5 fibroblasts up to 4 days after administration compared to conditioned medium of untreated controls. This proliferative effect was dependent on silica uptake as media from epithelial cells with cytochalasin D administration prior to silica addition failed to induce fibroblast proliferation (Figure 6A). Likewise, conditioned medium from NLRP3 siRNA transfected epithelial cells blunted higher DNA content levels in MRC-5 fibroblasts to baseline values (Figure 6B), indicating a role for the inflammasome in the initiation of neighboring fibroblast proliferation in response to silica. Conditioned media of LPS treated BEAS-2B cells did not affect fibroblast proliferation (Additional file 4: Figure S3).

## Discussion

In myeloid cells, the inflammasome receptor complex is recognized as one of the cornerstones of the long-sought intracellular surveillance system [20]. The NLRP3 (NALP3) complex contributes to innate immune defenses against pathogen- and danger-associated moieties such as exogenous or endogenous crystalline molecules [5-7,21].

Our present study assessed the importance of the bronchial epithelial cells as a target for cristobalite silica-induced inflammasome activation. Thereby, classical and non-canonical outputs of NLRP3 assembly and caspase-1 activation might contribute to the development of silicotic nodules and fibrosis. This study illuminated increases in transcription of components of the NLRP3 multiprotein platform which leads to caspase-1 activation in response to cristobalite silica in bronchial epithelial cells.

It has been postulated that the release of active IL-1 needs at first a signal provided by Toll-like receptor (TLR) engagement that results in gene transcription and pro-IL-1β accumulation, a process sometimes described as “priming”; and a second signal through NLR signaling, which results in caspase-1 activation and subsequently processing of this cytokine into mature bioactive IL-1β. Nevertheless, it is still controversial whether there is a direct role for TLR signaling in the activation of the inflammasome because of contrasting results regarding the requirement of a “two-hit-model” in the assembly of the multiprotein complex. Epithelial cell monolayers, like some myeloid cells, do not always require LPS priming in contrast with the well documented ‘two-hit-model’ and therefore may provoke a constant phase of alarm when bombarded with cristobalite particles. It is therefore not surprising to find only modest but significantly increased levels of the most potent pyrogen IL-1β, in whole cell lysates or medium of cell cultures after treatment with silica without priming with LPS. Although in the comparative experiments using THP-1 macrophages silica induced a 22-fold higher IL-1β release compared to the response in BEAS-2B cells, many more epithelial cells are present in the lungs compared to macrophages. Together with the observations that ambient particulate matter PM_10_ alone could induce increases of detectable cleaved subunits of IL-1β in bronchial epithelial cells [17] and described IL-1β release from bronchial epithelial cells after cigarette smoke and rhinovirus without priming with LPS [22,23], bronchial epithelial cells can be considered danger sensing cells contributing to small but relevant levels of biologically active IL-1β.

The nuclear-damage-associated molecular pattern molecule (DAMP), HMGB1 was originally identified as one of the nuclear proteins mediating gene transcription as a chromatin binder [24]. In secreted form, it is thought to be a cytokine-like molecule derived from various cell types, and acting as an alarmin. Furthermore, it induces production of pro-inflammatory cytokines [25]. Activated inflammasomes are involved in the release of HMGB1 through unconventional protein secretion, or as a soluble molecule from cells during pyroptosis [15]. HMGB1 is involved in innate and specific immune responses and contributes to acute lung injury in bleomycin-induced fibrosis in mice [26]. The same study also demonstrated that HMGB1 stimulates proliferation of human fibroblasts without effects on apoptosis and directly participated in fibrogenesis. Additionally, HMGB1 seems to protect the host from pathogen-induced mortality [27] and has the ability to attract stem cells to areas inflammation, thus promoting their regeneration [28,29]. Here we show that inflammasome activation mediates unconventional HMGB1 release from BEAS-2B and THP-1 cells following early interactions with silica and as a consequence further amplifying the reaction cascade.

bFGF is released after tissue injury and during inflammation and is produced by lung epithelial cells, macrophages and endothelial cells [30]. It plays a crucial role during fibrosis as well as angiogenesis [31,32]. We recently showed higher transcript levels and bFGF protein secretion in cristobalite silica treated BEAS-2B cells compared to untreated controls [18]. Here, we extended these findings by demonstrating inflammasome dependence of release of bFGF from epithelial cells and macrophages suggesting an early role for the inflammasome in initiating fibrogenesis.

In the previous study, we demonstrated uptake of cristobalite silica in bronchial epithelial cells [18]. Therefore, we investigated whether inflammasome activation was dependent on uptake of particles as shown by others in myeloid cells [5-7,21]. Decreased levels of bFGF and HMGB1 release occurred in medium after cytochalasin D pre-treatment, indicating a pivotal role for internalization of silica in inflammasome activation and release of pro-inflammatory and fibrotic mediators. It remains to be elucidated how particulates are internalized and how this triggers inflammasome assembly and activation.

Finally, possible effects of inflammasome activation in epithelial cells on the lung microenvironment were examined. It was found that lung epithelial cells after internalization of silica secrete substances, including bFGF that induce fibroblast proliferation *in vitro*. The fact that the inhibition of particle uptake and inflammasome inactivation in bronchial epithelial cells dramatically blunted fibroblast proliferative response is a novel observation of direct relevance to fibrogenesis.

**Figure 7:**
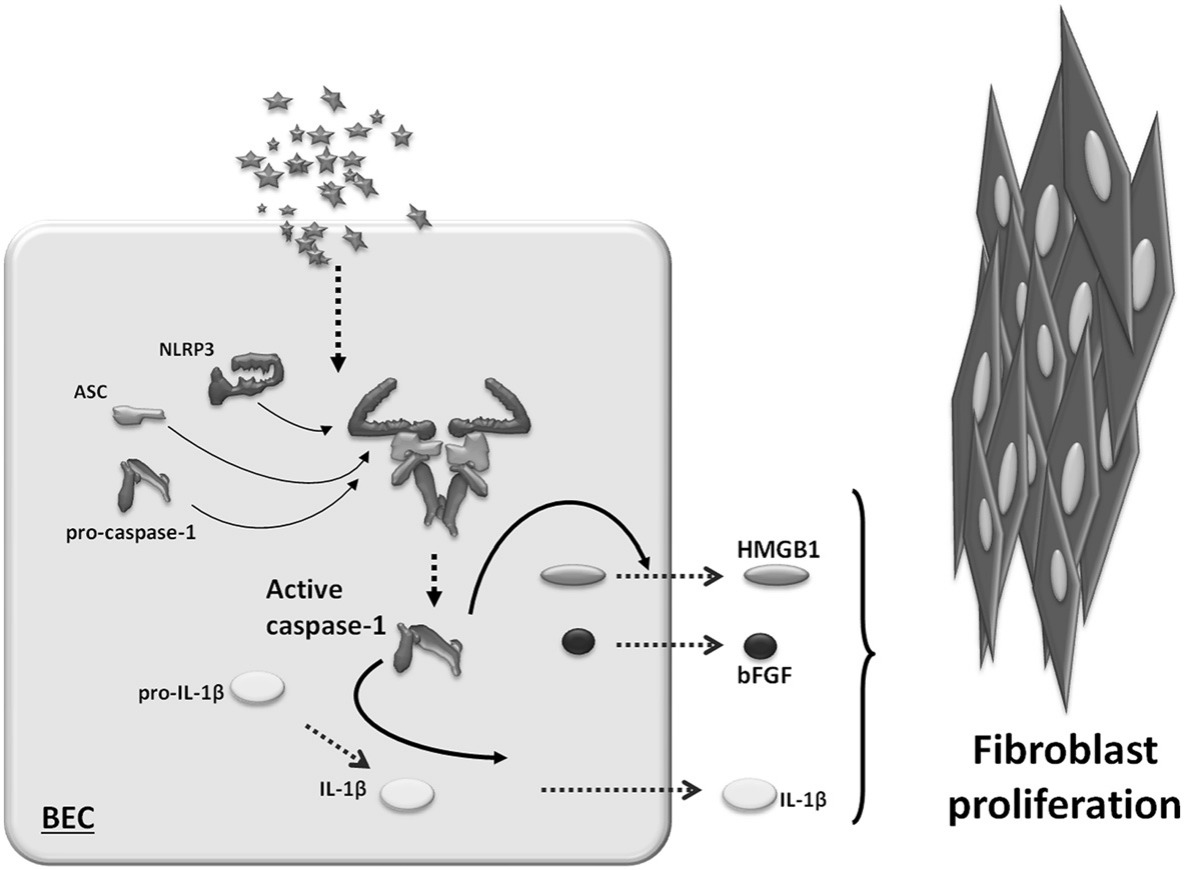
**A schematic representation of our *in vitro *model. **In bronchial epithelial cells (BEC), cristobalite silica particles initiate the production of inflammatory cytokines, alarmins and growth factors leading to fibroblast proliferation in a particle uptake and NLRP3 inflammasome-dependent manner.

The role of IL-1β in fibrosis is widely studied because IL-1β can induce its own gene expression and chronic activation via IL-1 receptor signaling. This could result in the continual cleavage of IL-1β via a positive feedback mechanism that could conceivably maintain a profibrotic phenotype. Uptake-related and inflammasome-dependent HMGB1 and bFGF secretion from lung epithelial cells suggest that crystalline silica induces local cellular injury which intensifies both disturbed immune signaling and increased proliferative capacity of cells during fibrogenesis (Figure 7).

Data presented here are the result of *in vitro* experiments and it remains to be investigated whether bronchial epithelial cells *in vivo* are a major site of silica-induced inflammasome activation and consequent signaling. Recently, it was published that human bronchial epithelial cells express functional NLRP3 [17] following PM_10_ exposure in a mouse model. Also, they demonstrated NLRP3-dependent airway neutrophilia and increases in dendritic cell numbers in intrathoracic lymph nodes, indicating the influence of NLRP3 on different aspects of immune cell signaling.

Collectively, our data indicate that bronchial epithelial cells are much more active than previously assumed. The NLRP3 inflammasome is activated in a one-step fashion, leading to downstream occurring events that are important in relaying the danger signal to the microenvironment contributing to the pathological condition of silicosis.

## Conclusion

These novel data show the presence and functional activation of the NLRP3 inflammasome by cristobalite silica in human lung epithelial cells that mediates a cadre of lung diseases. Moreover, these findings show that inflammasome activation by silica in epithelial cells is linked to fibroblast proliferation.

## Competing interests

The authors have no competing interests.

## Authors’ contributions

The study design was constructed by PMP, BTM, EFW and NLR. All experiments were performed by PMP. Additional cell culture, cell viability studies and ELISA experiments were performed by TNP. PMP performed the data analysis. PMP, BTM and NLR drafted the manuscript. All authors read and approved the final manuscript.

## Supplementary Material

Additional file 1: Figure S1 Assessment of BEAS-2B cell viability after exposure to silica particles for 24 h. Cell viability was assessed by the trypan blue exclusion assay. Results are expressed as the mean percent viable cells ± SEM compared to unexposed controls and are representative of 3 independent experiments (N = 3 in each experiment). Surface area concentrations and mass concentrations of particles are expressed as × 10^6^μm^2^/cm^2 ^and μg/cm^2 ^respectively. *represents p<0.05 compared to UC. Click here for file

Additional file 2: Table S1 Secreted levels of IL-1β, HMGB1 and bFGF from BEAS-2B and THP-1 cells in response to silica treatment measured by ELISA. Click here for file

Additional file 3: Figure S2 Silica-induced release of inflammatory mediators and DAMPs from THP-1 differentiated macrophages is NLRP3 dependent. ELISA performed on concentrated medium SN of PMA differentiated macrophages for detection of secreted IL-1β (**A**), HMGB1 (**B**) and bFGF (**C**) 24 h after silica treatment with or without priming with 5 μg/mL LPS for 4 hr. Data are presented as mean ± SEM with *p-value <0.05 compared to UC, # p-value <0.05 compared to the siControl group and $ p-value <0,05 compared to SIL150 alone. Click here for file

Additional file 4: Figure S3 Fibroblast proliferation is not affected by exposure to LPS-treated BEAS-2B conditioned medium. Click here for file
