# Supplementary figures and images for: Silica induces NLRP3 inflammasome activation in human lung epithelial cells
Source: Part Fibre Toxicol. 2013 Feb 12;10:3. doi: 10.1186/1743-8977-10-3 (PMC3607900; doi:10.1186/1743-8977-10-3)

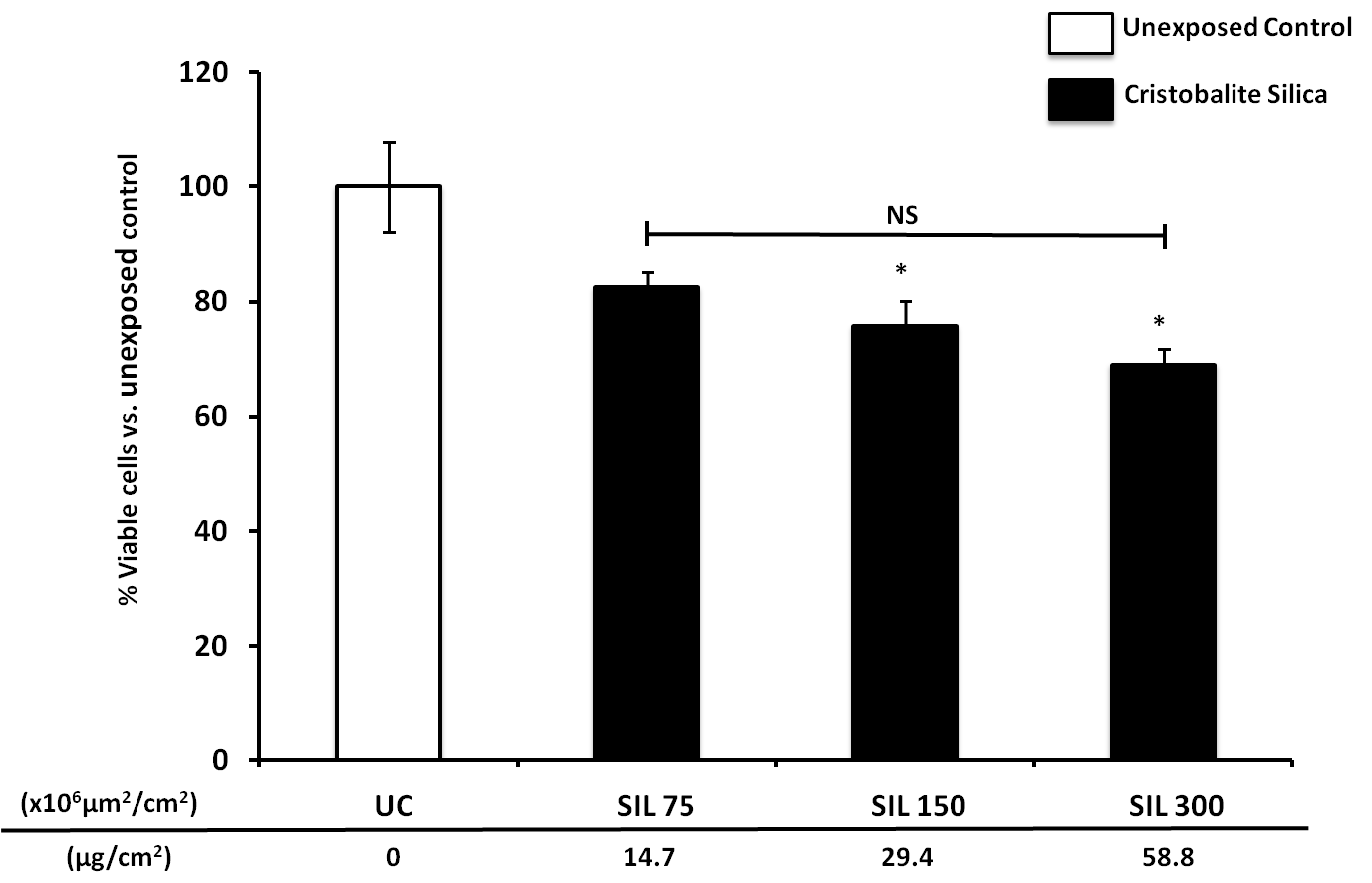

Supplement: Additional file 1: Figure S1 — Assessment of BEAS-2B cell viability after exposure to silica particles for 24 h. Cell viability was assessed by the trypan blue exclusion assay. Results are expressed as the mean percent viable cells ± SEM compared to unexposed controls and are representative of 3 independent experiments (N = 3 in each experiment). Surface area concentrations and mass concentrations of particles are expressed as × 106μm2/cm2 and μg/cm2 respectively. *represents p<0.05 compared to UC. [file 1743-8977-10-3-S1.tiff]

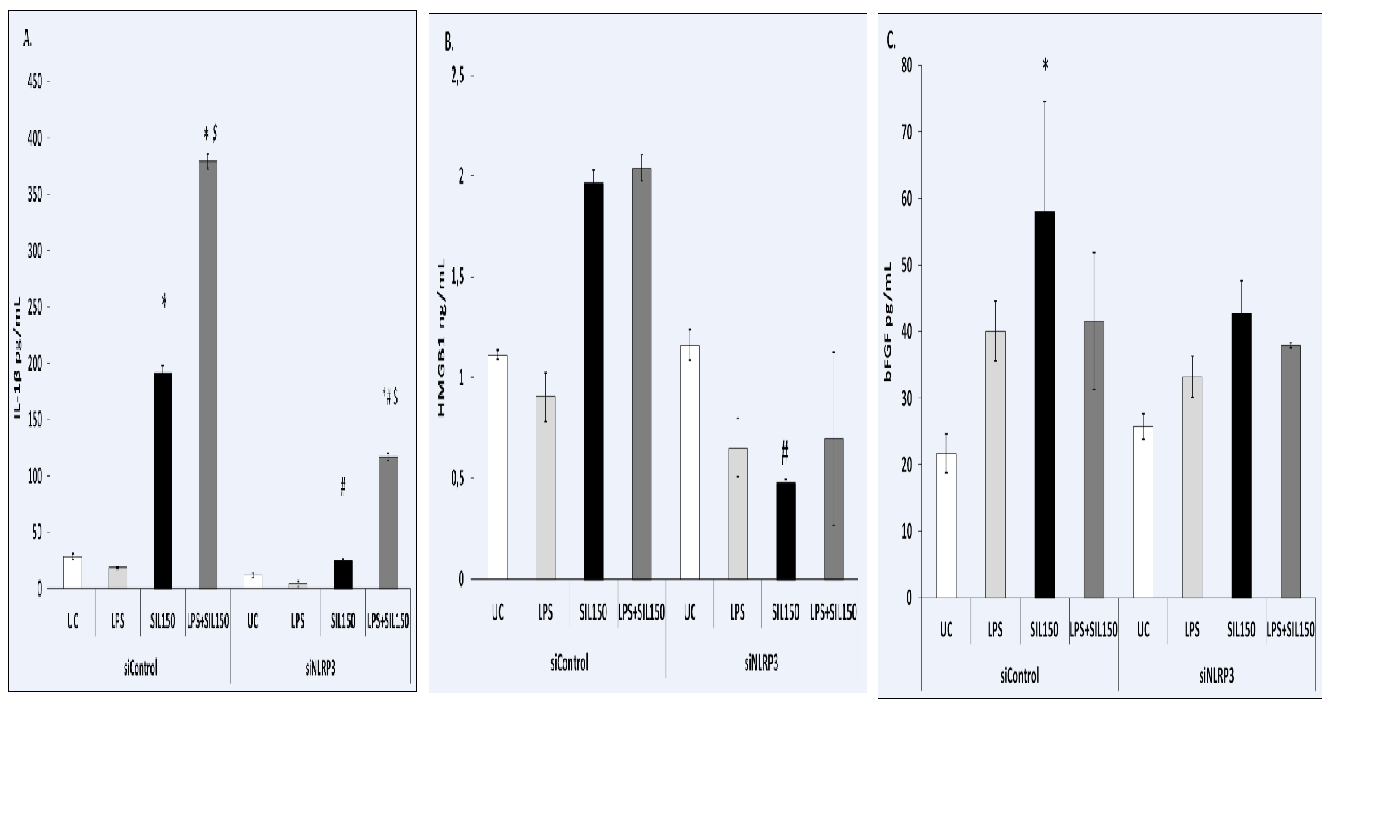

Supplement: Additional file 3: Figure S2 — Silica-induced release of inflammatory mediators and DAMPs from THP-1 differentiated macrophages is NLRP3 dependent. ELISA performed on concentrated medium SN of PMA differentiated macrophages for detection of secreted IL-1β (A), HMGB1 (B) and bFGF (C) 24 h after silica treatment with or without priming with 5 μg/mL LPS for 4 hr. Data are presented as mean ± SEM with *p-value <0.05 compared to UC, # p-value <0.05 compared to the siControl group and $ p-value <0,05 compared to SIL150 alone. [file 1743-8977-10-3-S3.tiff]

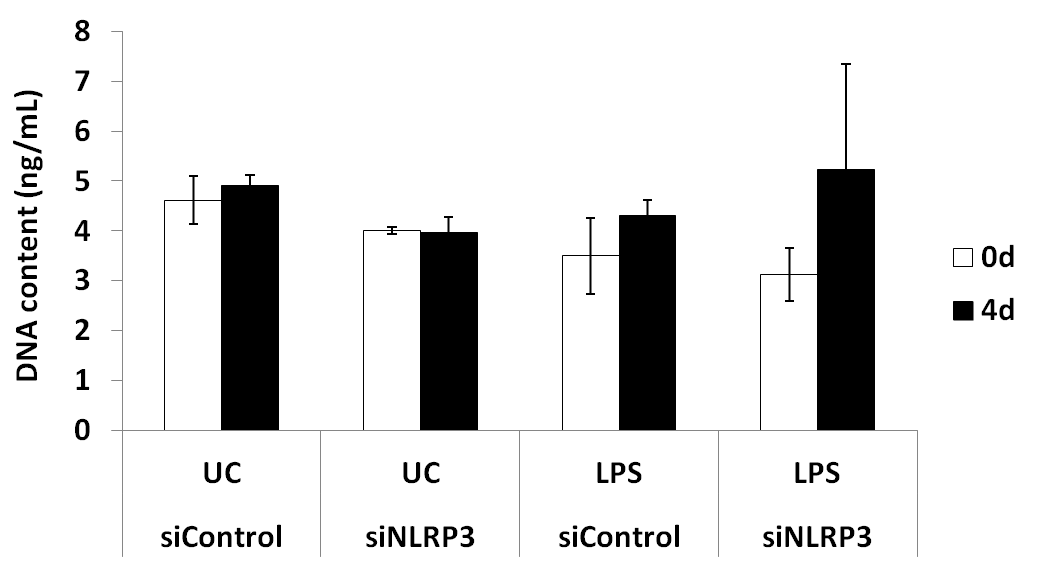

Supplement: Additional file 4: Figure S3 — Fibroblast proliferation is not affected by exposure to LPS-treated BEAS-2B conditioned medium. [file 1743-8977-10-3-S4.tiff]
