# Supplementary material for: Silica induces NLRP3 inflammasome activation in human lung epithelial cells
Source: Part Fibre Toxicol. 2013 Feb 12;10:3. doi: 10.1186/1743-8977-10-3 (PMC3607900; doi:10.1186/1743-8977-10-3)
Supplement: Additional file 2: Table S1 — Secreted levels of IL-1β, HMGB1 and bFGF from BEAS-2B and THP-1 cells in response to silica treatment measured by ELISA. [file 1743-8977-10-3-S2.docx]

**Supplemental Table**

Secreted levels of IL-1β, HMGB1 and bFGF from BEAS-2B and THP-1 cells in response to silica treatment measured by ELISA.

**
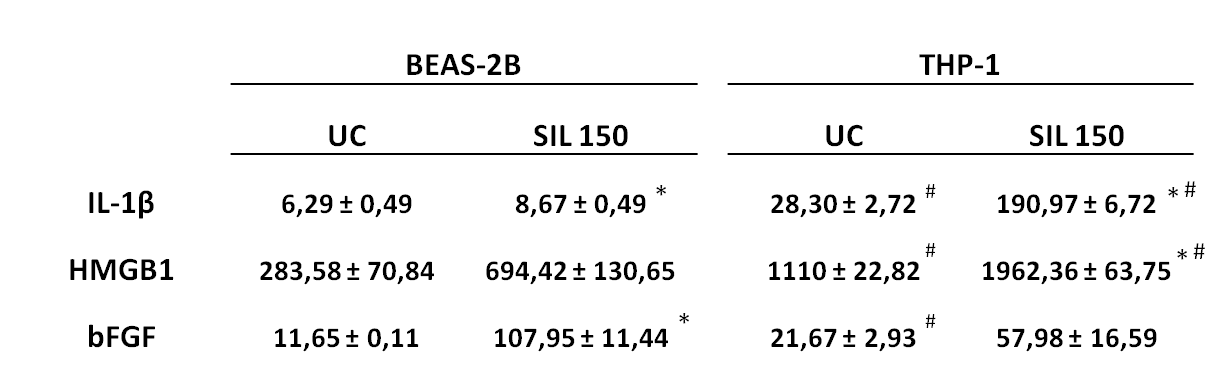
**

Data represented show IL-1β, HMGB1 and bFGF levels (pg/mL) under baseline conditions and after exposure to 150x10^6^µm^2^/cm^2^ cristobalite silica (SIL150) for 24h in BEAS-2B and THP-1 cells. * represents p-value < 0,05 for the difference between UC and SIL150 in each cell type. # represents p-value < 0,05 for the difference in concentration between both cell types.
